# Science Popularization Education regarding Oral Health-General Health for Nonmedical Undergraduates Applying a SPOC Teaching Model

**DOI:** 10.1155/2022/3439509

**Published:** 2022-07-01

**Authors:** Jingjing Luo, Ran Cheng, Lei Lei, Li Cheng, Yingming Yang, Tao Hu

**Affiliations:** State Key Laboratory of Oral Diseases, National Center of Stomatology, National Clinical Research Center for Oral Diseases, Frontier Innovation Center for Dental Medicine Plus, West China Hospital of Stomatology, Sichuan University, Chengdu, China

## Abstract

**Objective:**

To see how effective a blended teaching model based on a small private online course (SPOC) is in a science popularization education course on oral health-general health (OHGH).

**Methods:**

The SPOC blended teaching model was created using an elective classroom course “Oral Prophylaxis and Hygiene” in conjunction with an online learning course called “Preventive Dentistry” from the China University massive open online course (MOOC) for the science popularization education on OHGH. Students' evaluations and teaching efficacy of this science popularization education course were tested using pre- and postcourse questionnaires.

**Results:**

In all, 105 valid questionnaires were returned. Before the course, 95.2% of the students expressed an interest in learning more knowledge on oral disease and OHGH. When compared to those of the precourse, students' knowledge of oral diseases and OHGH was significantly higher (*P* < 0.0001) and the associated practice after science popularization education was much increased (*P* < 0.0001 or *P* = 0.0005), except for root canal therapy (*P* = 0.3886). The scores of students on the scientific popularization task also improved when compared to those of the previous classroom-only teaching (*P* < 0.0001). In the postcourse questionnaire, students rated the SPOC teaching mode significantly higher than both online learning and classroom teaching alone (*P* < 0.0001; *P* = 0.0117); the SPOC blended teaching was judged as more suitable for science popularization education (*P* < 0.0001).

**Conclusion:**

The application of the SPOC teaching mode for the science popularization education course on OHGH to nonmedical undergraduates has better teaching outcomes and is more likely to be accepted by college students.

## 1. Introduction

The World Health Organization has listed oral health as one of the ten criteria for human health, as a determinant indicator for the community's general health and well-being [1]. In dental clinical, the standard of oral health is “clean teeth, no carious cavity, no pain, normal gingiva color, no bleeding.” The Federation Dentaire Internationale (FDI) multifacetedly defined oral heath involving of available speaking, smiling, smelling, tasting, touching, chewing, swallowing, and a range of face-expressing emotions with confidence and with no pain, no discomfort, and no disease of the craniofacial complex [2]. Accompanied by increasing studies on the relationship between oral and systemic diseases, generally such as caries and cardia-cerebrovascular disease [3], periodontitis and diabetes or cardiovascular diseases [4, 5], and oral lichen planus and diabetes [6], it is commonly believed that oral health is an essential part of a person's general health and preventive dental care is essential for maintaining good health [7, 8].

Without solid background knowledge, it is usually stressful to make people other than medical research scholar or healthcare provider be aware of the association between oral health and systemic health. Since 2016, the Chinese Stomatological Association has used the topic “oral health, general health (OHGH)” as the focus of “Teeth-Care Day” to disseminate vital information to the general public in China, till now. Furthermore, policy documents [9, 10] have increasingly made it evident that strengthening oral health is vital for improving overall health, which necessitates the development of popular science. It also encourages the scientific spread of oral health knowledge from oral health practitioners across other groups, as well as oral health-general health awareness.

In the course “Oral Prophylaxis and Hygiene,” which is primarily for nonmedical undergraduates at Sichuan University [11], the teaching team insists on the concept of “prevention first” and teaches students about oral diseases and their correlation with systemic diseases through popularization, guiding them to understand the importance of maintaining oral health in order to achieve better overall health. This course has been taught in a typical classroom setting for the previous ten years. For nonmedical students, however, the knowledge of oral diseases and OHGH is difficult to apply, deepen, and integrate since it is abstract, scattered, and primarily remembered and because there are relatively few opportunities to participate in practice [12].

A small private online course (SPOC), in contrast to the massive open online course (MOOC) and other open online courses, has similar teaching resources as MOOC but is tailored to the needs of the participants, because of characteristics including a variety of delivery formats, high efficiency of dissemination, and high precision of the course content [13, 14]. Therefore, after trying some innovations in teaching methods such as online teaching, clinical case sharing, or guided discussions on the current public health situation as the COVID-19 outbreak [11, 15, 16], the SPOC blended teaching mode is further introduced in this course to better achieve an easy-to-understand but professional science popularization education for undergraduates, particularly for nonmedical undergraduates.

## 2. Materials and Methods

### 2.1. Participants and Study Design

The ethics committee of the West China Hospital of Stomatology at Sichuan University's Institutional Review Board approved this research (WCHSIRB-D-2018-092). Each participant signed an informed consent form. There were no deviations from the relevant protocols or rules. Undergraduates enrolled in a semester of the elective course “Oral Prophylaxis and Hygiene” in the fall semester of 2020–2021 at Sichuan University were selected as the study population. All participants submitted pre- and postcourse surveys. Students who submitted incomplete answers or showed contradictory answers were excluded. This semester's SPOC assignments were compared to those of the previous semester's traditional classroom teaching with the same topic.

### 2.2. Strategies of the SPOC Blended Teaching Model

Combining an online course called “Preventive Dentistry” from China University MOOC [17] with the classroom course “Oral Prophylaxis and Hygiene,” the SPOC blended teaching model was firstly created for the science popularization education on OHGH in this semester.

Each classroom teaching session began with authorization to launch the online learning platform. The teaching assistant assisted in emphasizing in knowledge points of oral diseases and OHGH, guiding students to form groups for self-directed learning, and creating online learning environments with online Q&A and discussion. Students were asked to fill out a questionnaire prior to the start of the course to determine their knowledge requirement. Before the survey, we reminded students that they were responsible for completing the survey on their own and the answers would not affect their final grade in the course. On the basis of the preclass questionnaire, we organize preclass discussion, focus on key topics in classroom teaching, and strive for high-quality interactive teaching activities that are based on the syllabus content as much as possible and oriented toward the requirements of students.

Aside from answering student questions and providing instruction for individualized learning, the teacher's primary role in classroom teaching is to finish the theoretical teaching of both important and difficult concepts. According to students' feedback, real-life case studies, drama simulations, the exchange of first-hand accounts of dental treatment by students, and animated video demonstrations were used to promote and popularize professional knowledge. At the same time, we help students integrate and assimilate the knowledge that they had learned both online and offline, as well as guided them to discuss and extend the dental knowledge that they had received from their own professional knowledge, as appropriate.

According to the students' classroom behavior, their comments, and summaries, teachers can use the process-oriented summary and feedback to improve future teaching programs and content. Students completed a postcourse questionnaire at the end of the semester as part of the multievaluation process for teaching. This semester's SPOC blended teaching was further compared to the prior semester only applying conventional classroom teaching, with the same teaching contents, to see if there is any difference in the final grades of science popularization assignments of students.

### 2.3. Pre- and Postcourse Questionnaires

The precourse survey consists of two parts. Basic information such as gender, year of study, and major is included in part 1 of the questionnaire, as well as the purpose of enrollment (for scores, for knowledge, and others). To complete part 2, students must answer 13 questions covering the following topics of course requirements: knowledge acquisition (caries, periodontal disease, malocclusion, pericoronitis of wisdom tooth, OHGH including caries and heart diseases, periodontitis and diabetes, malocclusion and jaw dysostosis, and pericoronitis of the wisdom tooth and miscarriage) and practice attempt (pit and fissure sealing, Bass brushing, fluoride in caries prevention, root canal therapy, and practice in science popularization).

In addition to the same 13 questions covering course requirements as the precourse questionaries, two teaching assessment items are included in the postcourse survey. These include scoring for the online learning content, classroom teaching content, and SPOC blended learning content, as well as the most likely choice of science popularization education. A universal questionnaire designer (http://www.wjx.com) was used to generate and manage both surveys.

### 2.4. Data Collection and Analysis

SPSS 25.0 (IBM Corp., New York, NY, USA) was used to get the Cronbach's alpha coefficient [11]. The data were analyzed by GraphPad Prism 9.1 software. One professor, three associate professors, and two lecturers evaluated the instrument's validity in terms of its clarity and conciseness [18]. The measurement data were expressed as “median (25% and 75% quartiles) [M(P25, P75)]” using the Mann–Whitney *U* test. The count data were tested with the chi-square (*χ*^2^) test. Statistically significant differences were set at *P* < 0.05.

## 3. Results

### 3.1. Nonmedical Students Preferred to Choose This Science Popularization Course for More Knowledge of Oral Science and OHGH

**Figure 1:**
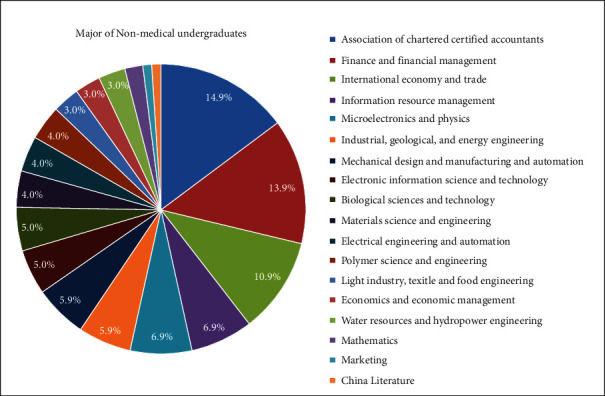
Major of nonmedical undergraduates. The top three were majors of the association of chartered certified accountants, finance and financial management, and international economy and trade.

At the end of survey, 105 out of 139 undergraduates (63 females and 42 males) completed both pre- and postcourse questionnaires and the answers were all valid. Most students (81.9%) taking this course were the third-year undergraduates, while there is no freshman. Additionally, while enrolling in this science popularization course, up to 96.2% were nonmedical undergraduates and 95.2% students wanted to gain a better understanding of oral diseases and OHGH, instead of getting scores. The information of participants and their purpose to select this course are shown in Table 1. All the 105 students were from 19 nonmedical majors of Sichuan University, and data are displayed in Figure 1.

### 3.2. Both Knowledge of Oral Diseases, OHGH, and Associated Practice of Students Was Improved, as well as Their Final Semester Scoring

The questionnaire items were given a content validity index (CVI) which included that the item-level CVI (I-CVI) and the scale-level (S-CVI) were both 1.0. Reliability analysis of the items was indicated by the Cronbach's alpha coefficient being 0.751 for the precourse survey and 0.766 for the postcourse survey.

Before and after the SPOC course, we examined the students' knowledge of common oral diseases (mainly caries, periodontal diseases, malocclusion, and pericoronitis of wisdom teeth) and OHGH-related knowledge (mainly including caries and heart disease, periodontitis and diabetes, malocclusion and jaw dysplasia, and pericoronitis of wisdom teeth and miscarriage). We discovered that after popularizing the abovementioned knowledge, students' understanding of them improved dramatically (*χ*^2^, all *P* < 0.0001). In comparison to their prior hands-on oral health practice, everyone's routine oral health prevention and treatment practices (except for root canal treatment, *χ*^2^, *P* = 0.3886) improved significantly following the course (Bass brushing and fluoride in caries prevention, *χ*^2^, *P* < 0.0001; pit and fissure sealing, *χ*^2^, *P* = 0.0005), as did the involvement in OHGH's science outreach practice (*χ*^2^, *P* < 0.0001). All details are shown in Table 2.

**Figure 2:**
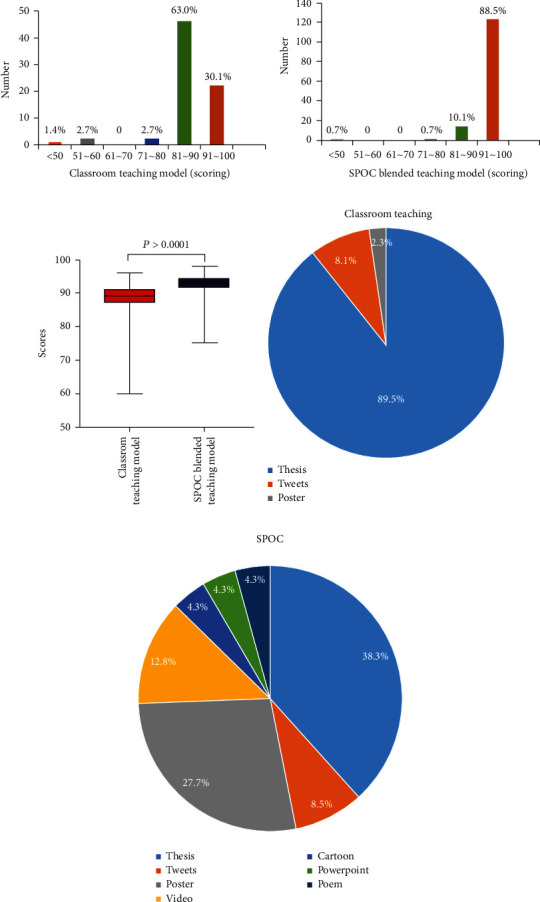
The final grades and forms of science popularization tasks in comparison to two semesters. (a, b) Distribution of students' grades in traditional classroom teaching and SPOC blended teaching during two semesters; (c) the comparison analysis between these two semesters; (d, e) distribution of students' task forms in traditional classroom teaching and SPOC blended teaching during two semesters.

Next, the final grades and task forms of students for science popularization tasks were compared between classroom learning and SPOC blended teaching using the equivalent textbooks, materials, syllabus, teaching hours, and instructors in both semesters. The study found that final scores for classroom learning were mostly in the range of 81–90 and those for SPOC blended training were primarily in the range of 90–100, with a statistically significant difference (*U* value = 1541, *P* < 0.0001, Figure 2). As for the forms, 89.5% of a student's popular science work, when taught according to the traditional classroom teaching, took the shape of a brief thesis or mini review. After taking the SPOC course, students have produced a wide range of popular science works, including posters (27.7%), videos (12.8%), tweets (8.5%), cartoons (4.3%), slides shows (4.3%), and poem (4.3%), besides a brief thesis (38.3%). Details were shown in Figure 2.

### 3.3. The SPOC Blended Teaching Model Was more Popular and Recommended by Students for Such Science Popularization Education

After students completed the entire science popularization education course, we analyzed their ratings of the online and classroom courses alone, as well as the SPOC blended teaching mode, and discovered that students gave the online course the lowest score, the classroom course the second highest, and the overall SPOC teaching mode the highest. The online and classroom modes of teaching were statistically significantly different from SPOC (online vs SPOC: *U* value = 3183, *P* = 0.0001; classroom vs SPOC: *U* value = 4477, *P* = 0.0117; details described in Table 3).

Throughout the course, students learned about various teaching methods and gained new insight into the scientific popularization education. In accordance with these findings, this study examined students' perceptions of the efficacy of different teaching methods in the science popularization course on oral diseases and OHGH. Students' evaluations indicated SPOC blended teaching to be more effective than online or classroom teaching alone in this science popularization education (online vs SPOC: *χ*^2^, *P* < 0.0001; classroom vs SPOC: *χ*^2^, *P* < 0.0001, details described in Table 4).

## 4. Discussion

Oral disease is no more merely a localized condition that affects the mouth; more and more research have revealed a tight link between oral disease and overall health [7, 8, 19–21]. Besides the well-established bidirectional relationship between periodontitis or oral lichen planus and diabetes mellitus [5, 6], the correlation between diabetes and peri-implantitis [22], a kind of periodontal or mucosal inflammation surrounding dentistry implant with pathogenesis remaining unclear [23], and denture stomatitis, a kind of oral mucosal fungal infection caused by wearing denture [24], has also been reported, although it is still somewhat controversial. In addition, some studies have reported that periodontitis could contribute to cancer metastasis [25], malocclusion had a close association with several systemic disorders such as pollinosis, food/drug allergy, and inhaled antigen allergy [19], and a variety of systemic diseases (e.g., Papillon-Lefèvre syndrome, mucocutaneous dyskeratosis, Coffin-Lowry syndrome, and Langerhans cell histiocytosis) was able to result in the loss of premature teeth in children at an early age [21]. In daily life, people are becoming more aware of the relationship between oral health and overall health, particularly in the context of the global COVID-19 pandemic, a major public health emergency, in which a variety of oral symptoms such as xerostomia and taste dysfunction are identified as its typical symptoms [26, 27].

With the “Health China 2030” initiative in place, China is working to improve both the country's general medical infrastructure and its citizens' understanding of how to take care of their bodies [28]. No doubt, this significant judgment has led to the acceleration of our science popularization education on oral health and its close correlation with general health. Education in health science popularization refers to the dissemination and improvement of the health quality of the public science system by making available to the general public the scientific knowledge, scientific spirit, scientific methods, and scientific ideas related to diseases and associated prevention and treatment that humans have acquired through the process of understanding and transforming nature [29]. People's desire for health science popularization information is increasing day by day as their economic status improves; at this point, health science popularization knowledge search has become the most significant type of web search for Chinese people and consumers in other countries [30, 31].

This research is based on the Oral Prophylaxis and Hygiene course in Sichuan University [11, 16], which is a high-quality public course aimed mostly at nonmedical undergraduates to distribute oral and general health information. Its ultimate goal is to aid college students and the society in gaining a better understanding of oral diseases and OHGH, as well as to guide the young generation in building the concept of humanistic care and the health concept of prevention before it arises. During the semester course in this study, 81.9% of participants were senior students implying that they had less demand for grades and better ability to accept some nonprofessional knowledge to learn medical knowledge. Moreover, 96.2% of students were not medical majors but they still expressed a strong desire to learn more knowledge about oral issues and OHGH. This desire increased significantly after the semester course. Additionally, following education, students' personal preventative oral healthcare activities [32, 33] such as pit and fissure sealing, Bass brushing, and fluoride in caries prevention rose dramatically. Given that root canal therapy is typically used to treat dental pulpitis or periapical inflammation [34, 35], the proportion of students who received this treatment did not differ significantly, implying that the oral health of the students in this study is better to some extent, despite the fact that 22.9% of the students received root canal treatment. The greatest benefit from this course was that the students were very receptive to the science popularization education of OHGH and they participated in or initiated relevant health science popularization actions from many angles after class.

Students are obliged to complete an OHGH-related popular science writing as a final exam at the end of each semester. Nearly all of student's popular science work, when taught according to the traditional classroom teaching, took the shape of a brief paper or mini review. Since taking the SPOC course, students have produced a wide range of popular science works, including videos, slide shows, and tweets, and their average grades have risen dramatically compared to the previous semester's classroom instruction, suggesting the SPOC's benefits as a teaching model. Aside from innovations in teaching methods, such as the introduction of MOOC [36], clinical case sharing, or discussion based on the current situation in real-time public health, there are still limitations in the previous classroom teaching, like as fixed teaching resources, single teaching format, and overly specific knowledge that impacts the attractiveness of this health science popularization education course to students and motivates their independent learning. SPOC is a more participatory and audience-specific type of online course, with features such as manageable scalability, resource customization, cross-course collaboration, and evaluation diversification [14, 37]. This semester of the OHGH science popularization education course is coupled with a MOOC of preventative dentistry [17], which is extremely suitable with the classroom teaching topic, hence modifying the teaching style to be “teacher-led and student-driven.” The SPOC teaching method was a stage success in this science education course, based on the findings of this study. It is also shown that the SPOC teaching model which is integrated online and by face-to-face learning was chosen by the most students in this sort of health science education course. As a result of this finding, it can be said that the SPOC teaching model can be better used in health science education. However, there are still a few students that do not like the SPOC teaching method. We'll need to perform further research to understand these outcomes and focus more on the limitation of a SPOC model.

## 5. Conclusions

As an old saying goes, providing a man with fish for one day is one thing; teaching him to fish is one thing that will provide him with food for the rest of his life. It is therefore underlined by the Chinese government that scientific innovation and innovation, along with scientific popularization, are the two pillars of achieving inventive development and that scientific popularization should be accorded the same importance as scientific and technological innovation. As the driving force of a thriving society, the health, values, and worldview of the youth are of the utmost significance. Using the science popularization education course of Prophylaxis and Hygiene at Sichuan University, this study proposed a blended teaching mode reform based on SPOC from which nonmedical students benefited. It facilitates their better understanding and application of oral health-general health knowledge and, to some extent, guiding the young generation to establish a positive oral health concept, a sense of social responsibility to maintain everyone's health, and a public health spirit. However, in this project, the small number of participants were involved and the limited OHGH knowledge and practices were explored. So more extensive, in-depth, and precise research is further needed to examine the most effective teaching models and methodologies for health science education.

## Figures and Tables

**Table 1:** Information of participants and purpose of enrollment.

Items	*N*	%
Gender		
Female	63	60
Male	42	40
Year of study		
1st year	0	0
2nd year	4	3.8
3rd year	86	81.9
4th year	15	14.3
Major		
Medical	4	3.8
Nonmedical	101	96.2
Purpose of enrollment		
For scores	4	3.8
For knowledge∗	100	95.2
Others	1	1

^∗^Both knowledge of oral diseases and OHGH.

**Table 2:** Gains of students after this science popularization education course.

Items		*N*		*P*
		Before	After	
Understanding of knowledge	Caries	53	105	<0.0001
	Periodontal diseases	45	91	<0.0001
	Malocclusion	13	78	<0.0001
	Pericoronitis of wisdom tooth	10	98	<0.0001
	Oral health-general health			
	Caries and heart diseases	17	75	<0.0001
	Periodontitis and diabetes	27	90	<0.0001
	Malocclusion and jaw dysostosis	61	100	<0.0001
	Pericoronitis of wisdom tooth and miscarriage	12	72	<0.0001

Practice after course	Pit and fissure sealing	12	36	0.0005
	Bass brushing	11	89	<0.0001
	Fluoride in caries prevention	6	42	<0.0001
	Root canal therapy	18	24	0.3886
	Practice in science popularization	3	91	<0.0001

**Table 3:** Scoring the teaching models in this science popularization education course.

Teaching contents	Scoring (0–10), *M* (*P*25, *P*75)	*P* ^∗^
Entire SPOC	9.0 (9.0, 10.0)	—
Online learning course	8.0 (7.0, 10.0)	<0.0001
Classroom teaching course	9.0 (8.0, 10.0)	0.0117

^∗^Comparison to the entire SPOC.

**Table 4:** Teaching model recommendation for science popularization education.

Items	*N*		
	Yes	No	*P* ^∗^
Classroom teaching	14	91	<0.0001
Online learning	3	102	<0.0001
SPOC blended teaching	88	17	—

^∗^Comparison to the entire SPOC.

## Data Availability

The data that support the findings of this study are available from the corresponding author upon reasonable request.
